# The “4.3,” A New Filler Lips Technique

**DOI:** 10.1111/jocd.16601

**Published:** 2024-10-24

**Authors:** Francesco Castellaneta, Mariagiovanna Lombardi, Santolo D'Antonio, Tommaso Fabrizio

**Affiliations:** ^1^ Department of Plastic and Reconstructive Surgery Basilicata Oncology Reference Center IRCCS‐CROB Rionero In Vulture Italy; ^2^ Department of Plastic and Reconstructive Surgery Federico II University of Naples Naples Italy

**Keywords:** filler, hyaluronic acid, lip rejuvenation, skin rejuvenation

## Abstract

**Background:**

The “4.3” technique is a hybrid lip augmentation approach that combines the aesthetic benefits of the vertical‐lip technique with the fewer entry points of the retrograde linear technique. This method aims to create natural‐looking, harmonious, and defined lips using hyaluronic acid (HA)‐based fillers, to enhance volume and contour.

**Aims:**

The objective of this study is to evaluate the effectiveness, safety, and reproducibility of the “4.3” technique in achieving natural lip augmentation with minimal filler volume and reduced injections. This technique is applied to improve the shape and volume of the lips while minimizing the risk of complications.

**Patients/Methods:**

The technique was applied to male and female patients with standard anatomical features who desired subtle enhancements in lip contour, projection, and volume. The procedure involved four injections in the upper lip and three in the lower lip, using a combination of retrograde linear and vertical techniques. A total of 0.7 mL of HA filler were used for each patient.

**Results:**

The “4.3” technique achieved consistent and aesthetically pleasing results across all patients, with improvements in lip volume, shape, and definition. The reduced number of injections minimized discomfort and risk of vascular complications. The use of Tri‐Hyal technology contributed to smooth, plump lips with harmonious proportions.

**Conclusions:**

The “4.3” technique is a standardized, intuitive, and reproducible method suitable for most patients, regardless of sex. It allows for natural‐looking lip augmentation using a reduced volume of filler and fewer injections, making it a safe and effective option for enhancing lip aesthetics.

## 1 Introduction

It is a hybrid technique that encompasses the aesthetic advantages of the vertical‐lip technique and the fewer entry points of the retrograde linear technique. The use of products from the Art Filler range (LABORATOIRES FILL‐MED), combined with the 4.3 technique, results in a natural‐looking, harmonious, hydrated, defined, rosy, and very pleasing appearance of the lips.

Two of the main fillers widely used are hyaluronic acid (HA) and polyacrylamide (PA) [1]. For lip enlargement through fillers, one can choose to inject the product into a single part or all anatomical areas of the lip, thus ensuring well‐manageable and predictable results. Among the injection techniques used for increasing lip volume are the serial puncture technique and linear threading, which can be performed in both anterograde and retrograde directions [2, 3, 4]. The decision to use a specific technique, or a combination of them, is guided by the desired aesthetic goals and the individual characteristics of the patient [5].

## 2 Patients and Methods

The technique is suitable for a wide range of patients, both men and women, with standard anatomical features who aspire to refine the contour of the upper and lower lip in the pale/vermilion zones, enhance the cupid's bow and the philtral columns for greater projection, increase the volume of the lips, both upper and lower, for a smooth and polished result, while maintaining a modest volume.

The shape and volume of the lips are enhanced through needle injections, which aim to gently lift the so‐called “White Lip Roll,” the lighter and softer tissue directly above the vermilion, thus optimizing the canine region of the lip arch without producing a “duck bill” effect. The shape of the philtrum can also be improved by treating the curvature of the lip and not directly injecting into the philtral columns.

## 3 Procedure

The procedure involves a combined needle injection technique, both retrograde linear and vertical, at four precise points corresponding to the upper lip and three on the lower lip. HA‐based fillers were used (Art Filler Lips with 0.3% Lidocaine). This filler, thanks to its Tri‐Hyal technology, allows for the achievement of the desired result. The first injection is made at the apex of the right cupid's bow and is guided medially toward the upper labial tubercle. Using the retrograde linear technique, a precise amount of hyaluronic acid (0.1 mL) is injected to add volume to the muscular plane of the lip, forming a columnar structure that serves to plump the tubercle and evert the upper lip. Through the same entry hole, the needle is then inserted laterally following the edge of the vermilion in a subdermal plane, where hyaluronic acid is released using the retrograde linear injection technique for better contour definition in that area (0.05 mL). Subsequently, the lateral portion is treated with an additional entry hole using the retrograde linear technique (0.05 mL). A mirror technique is used to complete the contralateral upper hemilip. In total, four injections are performed on the upper lips, injecting a total of 0.4 mL.

For the lower lip, only three injection points are used, the first at the edge of the vermilion on the midline (0.1 mL), and the other two at the midpoint of the two hemilips, again corresponding to the vermilion (0.05 mL are injected per injection site). Three injections are carried out on the lower lips, totaling 0.3 mL injected.

The injection involves a combined vertical and retrograde linear technique with art filler lips. It is important to ensure that the filler is not injected too superficially to prevent possible artifacts, nor too deeply, as this could lead to vascular complications.

The total base volume injected is 0.7 mL; the remaining 0.3 mL is used for subsequent touch‐ups according to the characteristics and needs of each patient.

## 4 Discussion

The 4.3 technique ensures the least possible invasiveness, resulting in reduced pain perception by the patient and a lower likelihood of vascular complications. The vascularization of the lips is provided by the superior and inferior labial arteries: These arteries originate from the facial artery, follow a path medially, reaching both the upper and lower lips. They branch out to supply blood to the mucosa and skin of the lips, as well as to the surrounding muscles [6, 7, 8]. It is crucial to identify the correct plane of injection, particularly in correspondence with the median line, where the labial artery is more frequently superficial [7, 9].

**FIGURE 1:**
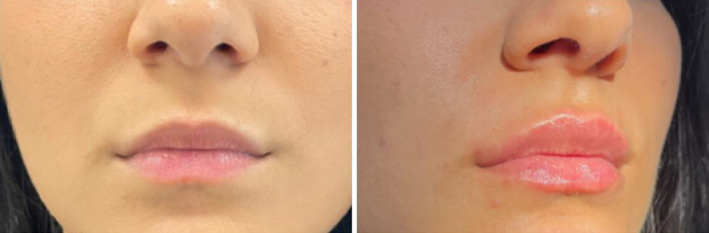
Case 1, before and immediately after the lip augmentation procedure with technique 4.3.

**FIGURE 2:**
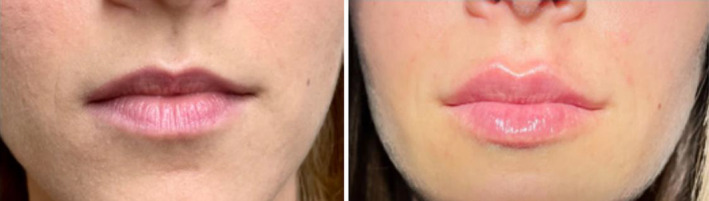
Case 2, before and immediately after the lip augmentation procedure with technique 4.3.

The use of a filler with Tri‐Hyal technology enables the achievement of well‐defined, plumped, smooth lips with a harmonious volume that is never excessive (Figures 1 and 2). The reduction in the number of injections and the reduced volume of filler used also translates into a lower possibility of formation of palpable or visible product nodules, filler accumulation, and the risk of bruising [10, 11, 12].

## 5 Conclusions

In conclusion, “4.3” is a “standardized,” simple, intuitive, and most importantly, reproducible technique that can be used in most patients, of both sexes, to achieve natural‐looking lips, reducing the volume of filler injected (the treatment involves the use of about 0.7 mL) and the number of injections the patient has to undergo.

## Conflicts of Interest

The authors declare no conflicts of interest.

## Data Availability

The data that support the findings of this study are available from the corresponding author upon reasonable request.
